# Comparison of SARS-CoV-2 Receptors Expression in Primary Endothelial Cells and Retinoic Acid-Differentiated Human Neuronal Cells

**DOI:** 10.3390/v13112193

**Published:** 2021-10-30

**Authors:** Francesca Benedetti, Giovannino Silvestri, Carla Mavian, Matthew Weichseldorfer, Arshi Munawwar, Melanie N. Cash, Melissa Dulcey, Amy Y. Vittor, Massimo Ciccozzi, Marco Salemi, Olga S. Latinovic, Davide Zella

**Affiliations:** 1 Institute of Human Virology and Global Virus Network Center, Department of Biochemistry and Molecular Biology, School of Medicine, University of Maryland, Baltimore, MD 21201, USA; fbenedetti@ihv.umaryland.edu (F.B.); amunawwar@ihv.umaryland.edu (A.M.); 2 Institute of Human Virology and Global Virus Network Center, Department of Medicine, School of Medicine, University of Maryland, Baltimore, MD 21201, USA; gsilvestri@ihv.umaryland.edu; 3 Emerging Pathogens Institute, Department of Pathology, Immunology and Laboratory Medicine, College of Medicine, University of Florida, Gainesville, FL 32610, USA; cmavian@ufl.edu (C.M.); mcash@pathology.ufl.edu (M.N.C.); 4 Institute of Human Virology and Global Virus Network Center, Department of Microbiology and Immunology, School of Medicine, University of Maryland, Baltimore, MD 21201, USA; mweichseldorfer@ihv.umaryland.edu; 5 Emerging Pathogens Institute, Department of Environmental and Global Health, College of Medicine, University of Florida, Gainesville, FL 32610, USA; dulceym@ufl.edu (M.D.); Amy.Vittor@medicine.ufl.edu (A.Y.V.); 6 Medical Statistic and Molecular Epidemiology Unit, University of Biomedical Campus, 00128 Rome, Italy; m.ciccozzi@unicampus.it

**Keywords:** SARS-CoV-2, ACE-2, SARS-receptors, neuronal cells, endothelial cells

## Abstract

SARS-CoV-2 (Severe Acute Respiratory Syndrome Coronavirus 2) is primarily responsible for coronavirus disease (COVID-19) and it is characterized by respiratory illness with fever and dyspnea. Severe vascular problems and several other manifestations, including neurological ones, have also been frequently reported, particularly in the great majority of “long hauler” patients. SARS-CoV-2 infects and replicates in lung epithelial cells, while dysfunction of endothelial and neuronal brain cells has been observed in the absence of productive infection. It has been shown that the Spike protein can interact with specific cellular receptors, supporting both viral entry and cellular dysfunction. It is thus clear that understanding how and when these receptors are regulated, as well as how much they are expressed would help in unveiling the multifaceted aspects of this disease. Here, we show that SH-SY5Y neuroblastoma cells express three important cellular surface molecules that interact with the Spike protein, namely ACE2, TMPRSS2, and NRP1. Their levels increase when cells are treated with retinoic acid (RA), a commonly used agent known to promote differentiation. This increase matched the higher levels of receptors observed on HUVEC (primary human umbilical vein endothelial cells). We also show by confocal imaging that replication-defective pseudoviruses carrying the SARS-CoV-2 Spike protein can infect differentiated and undifferentiated SH-SY5Y, and HUVEC cells, although with different efficiencies. Neuronal cells and endothelial cells are potential targets for SARS-CoV-2 infection and the interaction of the Spike viral protein with these cells may cause their dysregulation. Characterizing RNA and protein expression tempo, mode, and levels of different SARS-CoV-2 receptors on both cell subpopulations may have clinical relevance for the diagnosis and treatment of COVID-19-infected subjects, including long hauler patients with neurological manifestations.

## 1. Introduction

As of August 2021, Coronavirus Disease 2019 (COVID-19) caused by SARS-CoV-2 has resulted in more than 210 million confirmed cases and about 4.5 million deaths worldwide (https://covid19.who.int/ accessed on 31 August 2021). The lungs are the primary target of viral infection and replication [1,2], though several studies have demonstrated the presence of viral genomes in other organs, including the pharynx, heart, liver, brain, and kidneys [3,4,5]. Accordingly, COVID-19 patients primarily experience respiratory illness with fever and dyspnea, followed by a number of other manifestations including gastrointestinal, olfactory, cardiovascular, and neurological manifestations [6,7].

Angiotensin-converting enzyme 2 (ACE2) is the major receptor that mediates the SARS-CoV-2 infection of target cells following the cleavage and activation of its envelope Spike protein by the transmembrane protease serine 2 (TMPRSS2) [8,9,10,11]. ACE2 has been detected in several tissues, including lungs and neurons [12,13,14,15]. Another molecule that facilitates viral entry is Neuropilin-1 (NRP1), a member of the neuropilins family of transmembrane receptors, which plays a key role in facilitating SARS-CoV-2 entry by binding furin-cleaved substrates [16,17]. However, a precise knowledge on how these different receptors and entry-facilitators are regulated in different tissues of the host is lacking.

SARS-CoV-2 RNA has also been detected in the brain tissue of deceased patients [4] and several in vitro experiments have previously shown that SARS-CoV-2 infects cell brain cultures [18,19,20], though productive in vivo infection has not been convincingly demonstrated yet. Neurological symptoms, including headache, anosmia, ageusia, insomnia, confusion, seizure, and encephalopathy, have been frequently reported in about 40% to 85% of hospitalized COVID-19 patients [3,21]. Overall, these data point to a potentially important involvement of SARS-CoV-2 in causing effects on the mental health of SARS-CoV-2 survivors [22]. Autopsy studies on COVID-19 patients have also suggested the presence of viral particles in the vascular beds of different organs [23,24] and others have shown SARS-CoV-2 infection of cultured endothelial cells [25,26]. Notably, some COVID-19 patients also experience vascular inflammation, barrier defects leading to tissue edema, and activation of disseminated intravascular coagulation and microthrombi. Indeed, vascular events and dysfunction of endothelial cells are major complications of COVID-19. Moreover, pre-existing impaired endothelial cells’ functions, such as those observed in diabetes mellitus patients and those underlying vascular pathologies, are associated with worsening clinical progression [24,27,28,29,30,31,32]. Systemic inflammatory response, either as a direct consequence of the viral infection or as an indirect effect triggered by virus–cell interaction, has been proposed to account for these effects, though the contribution of each mechanism is unclear.

To study how SARS-CoV-2 could interact with both the brain and vascular endothelial system, we used two broadly used cell types, namely a human neuroblastoma cell line (SH-SY5Y) and the human umbilical vein endothelial cells (HUVEC).

The SH-SY5Y cell line, a subline of SK-N-SH cells, was established in the early 1970s from a bone marrow biopsy of a neuroblastoma patient [33]. In its undifferentiated state, this stem-like cell line is composed of a relatively homogeneous neuroblast-like cell type (N type) derived from immature neoplastic neural crest cells. Upon differentiation, SH-SY5Y cells exhibit several biochemical and functional properties of neurons, including biochemical, ultrastructural, morphological, and electrophysiological similarity, as well as several neuronal-specific markers [34,35]. For this reason, it has been broadly used as an in vitro model to characterize biological properties and responses of neuronal cells since the early 1980s [36]. The SH-SY5Y cells possess the capability of proliferating in long-term culture and treatment with a variety of agents is used to differentiate them, such as phorbol ester 12-O-tetradecanoylphorbol-13-acetate (TPA) [37], the brain-derived neurotrophic factor (BDNF) [38], dibutyryl cyclic AMP (dBcAMP) [39], purine [40] or staurosporine [41], and retinoic acid (RA) [42]. In particular, the effect of RA on the differentiation towards a mature cholinergic phenotype [43] through the regulation of several cellular pathways, including the transcription of neurotrophin receptor genes [44], the Wnt-signaling pathway [45], and pathways involving type II protein kinase A (PKA) [46], is well characterized.

More specifically, SH-SY5Y are differentiated from a neuroblast-like state into mature neurons by RA treatment. SH-SY5Y cells proliferate very rapidly and appear to be non-polarized with very few and short processes while in an undifferentiated status. They also grow in clumps and their surface markers are indicative of immature neurons. Additionally, differentiated SH-SY5Y show long and branched processes, their proliferation rate decreases, and they express different markers and proteins resembling mature neurons [47,48]. Once differentiated into mature neurons, SH-SY5Y can be maintained for up to 2 weeks post-terminal differentiation and used for experimentation [48]. It is well known that RA-differentiated SH-SY5Y cells provide the closest approximation of mature human neurons found in vivo and treatment with RA is the most widely used as well as accepted differentiation method protocol [47,49,50]. These differentiated SH-SY5Y cells thus provide a well-established and advantageous model for the characterizations of their neurobiology, for the study of neurotropic viruses, and for the screening of chemotherapeutic toxicity in neurons [48,51].

HUVECs provide a classic model system to study many aspects of endothelial function and disease. Endothelial cells are, in fact, major participants and regulators of inflammatory reactions. Additionally, endothelial cells play a role in the development and maintenance of neuronal function and plasticity, including involvement in neuropathological conditions [52,53]. In particular, SARS-CoV-2 was shown to infect vascular organoids in vitro [54] and some groups reported endothelial infection in both glomerular capillary loops and skin lesions [24,28,55]. SARS-CoV-2-infected endothelial cells also play a key role in sustaining the inflammation, aberrant angiogenesis, and chemoattraction of immune cells in the early phases of the viral infection [15]. However, ACE2 expression is low in endothelial cells, hence these cells may not be the primary target of SARS-CoV-2 in the vascular wall [56]. Nonetheless, it has been shown that the proper function of the endothelium might also be affected by indirect viral action caused by the Spike viral protein, most likely interacting with cellular co-receptors, though the precise mechanism(s) have not been fully clarified yet [57].

Here, we analyze the expression of different cellular molecules responsible for SARS-CoV-2 infection in two cell types, namely HUVEC and SH-SY5Y, both undifferentiated and RA-differentiated. Endothelial cells, in fact, interact with all the different types of neuronal cells by regulating molecular and cellular trafficking, including the circulation of viruses. This is why investigating their activation, which can lead to viral invasion, is so important to describe and fully understand the neurological manifestations in “long hauler” patients. By comparing their expression both at the protein and mRNA levels, we showed that the differentiation of neuronal cells slightly affects ACE2 expression, while NRP1 and TMPRSS2 membrane detection is noticeably increased and reaches high levels as measured on HUVEC cells. By characterizing their expression, we also assessed their ability to support viral entry mediated by the SARS-CoV-2 Spike protein. Our data may be relevant for a better understanding of the molecular mechanisms leading vascular pathologies and neurological symptoms associated to long-COVID-19.

## 2. Results

### 2.1. RA-Differentiation of SH-SY5Y Slightly Increases ACE2 Receptor Expression

**Figure 1:**
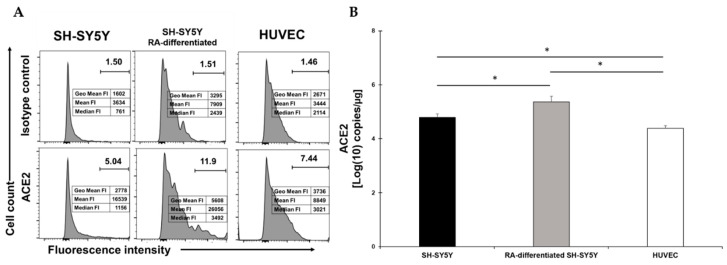
(**A**) Flow cytometry analysis of ACE2 expression in SH-SY5Y (left), RA-differentiated SH-SY5Y (middle), and HUVEC (right) cells. Both SH-SY5Y and HUVEC cells were stained with the specific antibody for the receptor (bottom) or the corresponding isotype control (top). The data related to the fluorescent intensity are also indicated in the figure. A representative of three different experiments is shown. (**B**) mRNA expression levels of ACE2 in both SH-SY5Y and RA-differentiated SH-SY5Y and HUVEC cells. Experiments were done in duplicates. Copy number per µg of mRNA is indicated. * *p* < 0.05.

We first determined the presence of the most important membrane molecule involved in SARS-CoV-2 entry, namely ACE2. We performed a flow cytometry assay on undifferentiated SH-SY5Y neuronal cells and compared its presence on the surface of RA-differentiated SH-SY5Y cells (Figure 1A). As previously described [43], RA-treatment resulted in the differentiation of SH-SY5Y cells, as demonstrated by the appearance of long axons (cfr. in Figure S1A–D). To better quantify the presence of ACE2 on the surface, we also calculated the fluorescence intensity (Figure 1A). For comparison, the ACE2 was also measured on HUVEC. While a very small percentage of both SH-SY5Y and HUVEC expressed ACE2, the percentage clearly increased with RA-differentiation (Figure 1A). RA-differentiation not only increased the percentage of ACE2-postive cells but also increased the presence of the receptors, as assessed by the fluorescent intensity (Figure 1A). Quantification of mRNA allowed us to confirm these data (Figure 1B). ACE2 expression was significantly higher in RA-differentiated SH-SY5Y compared to the undifferentiated cells, while ACE2 expression was significantly lower in HUVEC cells compared to both types of neuronal cells (Figure 1B).

### 2.2. TMPRRS2 Surface Expression Is Upregulated in SH-SY5Y following RA-Differentiation

**Figure 2:**
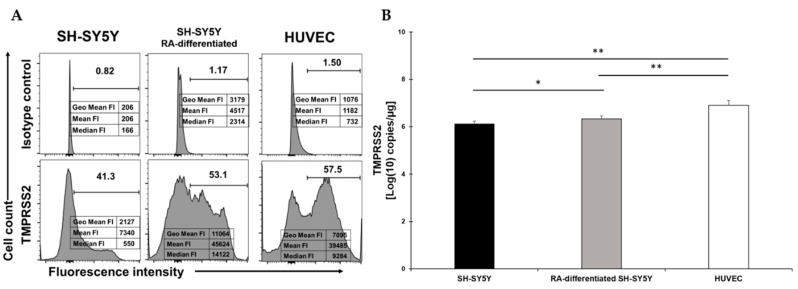
(**A**) Flow cytometry analysis of TMPRSS2 expression in SH-SY5Y (left), RA-differentiated SH-SY5Y (middle), and HUVEC (right) cells. Both SH-SY5Y and HUVEC cells were stained with the specific antibody for the receptor (bottom) or the corresponding isotype control (top). The data related to the fluorescent intensity are also indicated in the figure. A representative of three different experiments is shown. (**B**) mRNA expression levels of TMPRSS2 in both SH-SY5Y and RA-differentiated SH-SY5Y and HUVEC cells. Experiments were done in duplicates. Copy number per µg of mRNA is indicated. * *p* < 0.05 and ** *p* < 0.01.

TMPRSS2 was clearly present on about 40% of SH-SY5Y cells and this percentage slightly increased following RA-differentiation, even though it did not reach the percentage observed in HUVEC (Figure 2A). The fluorescent intensity indicated a higher signal in RA-differentiated neuronal cells, suggesting an enhanced presence higher than what was observed in HUVEC (Figure 2A). The mRNA quantification showed a statistical difference between treatment and no treatment, but in both cases, TMPRRS2 expression was significantly lower than the levels observed in HUVEC, even though the surface levels were higher (Figure 2B). These levels were not reflected by the measurement of the fluorescent intensity (cfr. in Figure 2A,B), indicating that some post-transcriptional mechanism is responsible for the differences observed.

### 2.3. RA-Differentiation Increases Levels of the NRP1 Surface Receptor in SH-SY5Y Cells

**Figure 3:**
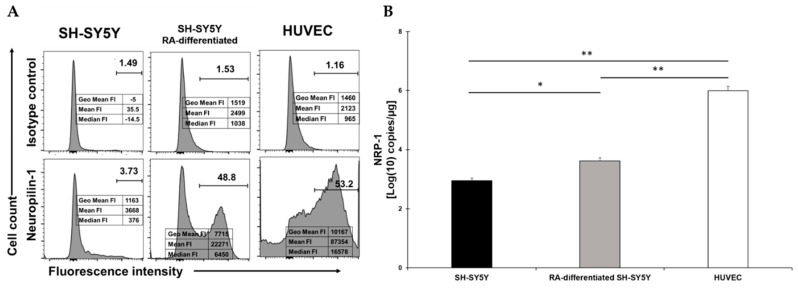
(**A**) Flow cytometry analysis of NRP1 expression in SH-SY5Y (left), RA-differentiated SH-SY5Y (middle), and HUVEC (right) cells. Both SH-SY5Y and HUVEC cells were stained with the specific antibody for the receptor (bottom) or the corresponding isotype control (top). The data related to the fluorescent intensity are also indicated in the figure. A representative of three different experiments is shown. (**B**) mRNA expression levels of NRP1 in both SH-SY5Y and RA-differentiated SH-SY5Y and HUVEC cells. Experiments were done in duplicates. Copy number per µg of mRNA is indicated. * *p* < 0.05 and ** *p* < 0.01.

NRP1 was expressed at low levels in SH-SY5Y cells and dramatically increased following RA-differentiation (Figure 3A). However, while the percentage of SH-SY5Y-positive cells were similar to HUVEC, the reduction in the fluorescent intensity indicated that less receptor molecules were present (Figure 3A). The mRNA quantification showed an increase of NRP1 expression in RA-differentiated cells and HUVEC. The mRNA quantification showed a statistical increase following RA-differentiation of SH-SY5Y cells and an even higher level of mRNA in HUVEC, and these data matched the levels of NRP1 as observed on the surface (Figure 3B).

### 2.4. RA-Treatment of SH-SY5Y Markedly Increases Spike-Mediated SARS-CoV-2 Entry

Next, we assessed whether the increased detection of surface receptors resulted in enhanced susceptibility to SARS-CoV-2 Spike-mediated cellular entry. For this purpose, we utilized a pseudovirus expressing the Spike of SARS-CoV-2 and carrying the D614G-mutated Spike (PV-SARS-CoV-2-S-(D614G)-VSV-△G-mCherry). Upon entry into the host’s cells, the pseudovirus expresses a red color chemo-luminescent protein that is then detectable by confocal microcopy. Since it does not allow for viral replication, this system is well suited for studying the first steps of viral fusion and entry.

**Figure 4:**
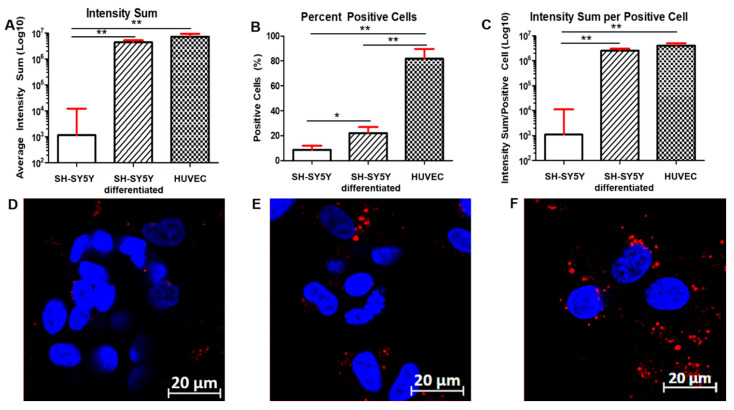
Entry of PV-SARS-CoV-2-S-(D614G)-VSV-△G-mCherry in SH-SY5Y (**A**–**D**), RA-differentiated SH-SY5Y cells (**B**–**E**), and HUVEC cells (**C**–**F**). Upon infection with the pseudovirus carrying the Spike with the D614G mutation, images were acquired after 24 h post-infection. The average intensity sum of the signal (**A**), the number of positive cells (**B**), and the average intensity sum of the signal/positive cell (**C**) were calculated for both SH-SY5Y and RA-differentiated SH-SY5Y and HUVEC cells. Results represent the mean and SEM of 10 different locations in each well. Representative images of SH-SY5Y cells (**D**), RA-differentiated SH-SY5Y cells (**E**), and HUVEC cells (**F**) show the viral entry. DAPI-staining was used for nuclei detection. Scale bar: 20 µm. * *p* < 0.05 and ** *p* < 0.01, calculated using a two-tailed Student’s *t*-test. A representative of three different experiments is shown.

When the SH-SY5Y cells were infected with the lentivirus, we observed an increase in susceptibility to Spike-mediated entry in RA-differentiated cells, as compared to undifferentiated cells (Figure 4). The average intensity of the signal (about a 6.5 times increase), the percentage of positive cells (about a 2.5-fold increase, 8.7% in SH-SY5Y cells versus 22.1% in differentiated SH-SY5Y cells), and the intensity of the signal/positive cells were also higher in RA-differentiated SH-SY5Y cells (about a 4.2-fold increase; Figure 4A–C). Representative images of non-differentiated (Figure 4D) and RA-differentiated SH-SY5Y cells (Figure 4E) show clear differences in the intensity of the red signal, where red signals display the Spike protein expressed in the target cells, while blue ones display the nuclei of the infected cells (Figure 4D,E). Uninfected cells, neither undifferentiated nor RA-differentiated, did not show any background signal (Figure S2A,B). In order to gain better visual support and to validate the pseudoviral particles’ presence within the cells, we proceeded with the z-stack acquisition (and ortho mode) of the differentiated cells. The distribution of the pseudoviral particles in the RA-stimulated cells is shown in Figure S3A–C).

HUVECs appeared to be susceptible to Spike-mediated entry as much as the differentiated SH-SY5Y cells when infected with the pseudovirus (Figure 4). In fact, we did not observe any statistical difference between differentiated SH-SY5Y cells and HUVECs when we calculated both the average intensity of the signal and the intensity of the signal/positive cells (Figure 4A,C). However, we observed a higher percentage of HUVEC-positive cells (81.7%) compared to both SH-SY5Y and differentiated SH-SY5Y cells (Figure 4B). A representative image of HUVEC cells (Figure 4F) infected with the pseudovirus is shown (Figure 4F). Uninfected HUVECs did not show any background signal (Figure S4). Additionally, in this case, we proceeded with the z-stack acquisition (and ortho mode) of the cells to gain better visual support and to validate the pseudoviral particles’ presence within the cells. The distribution of the pseudoviral particles in HUVECs is shown in Figure S5A–C.

Overall, our data clearly indicate that RA-differentiated SH-SY5Y neuroblastoma cells show an increased expression of the surface molecules used by SARS-CoV-2 to enter the cells, namely NRP1, TMPRSS2, and, to a lesser extent, ACE2. In all cases, the data were confirmed by quantification of mRNA. The result is an increased entry of the pseudovirus into the cells, as demonstrated by confocal microscopy. In HUVEC cells, we observed even higher expression of NRP1 and TMPRSS2 compared to both types of neuronal cells, while ACE2 was the least expressed. Both neuronal cells and endothelial cells are potential target for SARS-CoV-2 infection and the interaction of the Spike viral protein with these cells may cause their dysregulation. Comparison with HUVEC cells indicated that ACE2 was slightly higher in differentiated neuronal cells in terms of cells’ percentage positivity (11.9 in RA-SH-SY5Y versus 7.44 in HUVEC) and intensity; TMPRSS2 was higher in intensity but slightly lower in cells’ percentage positivity (53.1 in RA-SH-SY5Y versus 57.5 in HUVEC); and NRP1 was slightly lower in cells’ percentage positivity (48.8 in RA-SH-SY5Y versus 53.2 in HUVEC) and intensity. The receptor expression of ACE2 NRP1, and TMPRSS2 was confirmed by mRNA quantification. Regarding the viral entry, HUVEC cells appeared to be as susceptible as differentiated SH-SY5Y cells. The only difference concerned the number of positive cells infected, which was higher in the HUVEC cells’ culture.

## 3. Discussion

Respiratory illness following SARS-CoV-2 infection of the lung cells is the hallmark symptom of COVID-19, though many other tissues are targeted by the virus [1,2]. In this regard, it is worth noting the clinical severity and cellular damage ensuing in both the brain and vascular system as consequence of infection.

A number of recent studies have shown the ability of SARS-CoV-2 to infect neuronal cells cultured in vitro [18,19,58,59,60]. Moreover, in light of the significant neurological and psychiatric outcomes in COVID-19 survivors [61], understanding how SARS-CoV-2 receptors are regulated on neuronal cell subpopulations would shed light on how cellular and brain functions may be affected by viral entry and potential replication. SARS-CoV-2 may also exert its negative effect by merely interacting with target cells and thus dysregulating important cellular pathways. Indeed, it has been shown that the proper function of the endothelium might also be affected by indirect viral action caused by the Spike viral protein, most likely interacting with cellular co-receptors, though the precise mechanism(s) have not been fully clarified yet [57]. It is worth noting that only HCAEC (primary human coronary artery endothelial cells) has been shown to express the SARS-CoV-2 receptor ACE2, which is required for virus infection. Accordingly, infection with the SARS-CoV-2 variants B.1.1.7 (alpha variant), B.1.351 (beta variant), and P.2 (zeta variant) resulted in significantly higher levels of the viral Spike protein. Despite this, no intracellular double-stranded viral mRNA was detected and the supernatant did not contain an infectious virus. It thus appears that certain cellular receptors play a key role in cell susceptibility to infection and in causing the possible negative effect that they can deliver to the cell when improperly engaged by the virus Spike protein.

For this reason, the regulation of SARS-CoV-2 co-receptors in target tissues is being actively investigated. For example, it is known that ACE2 and TMPRSS2 expression in subsets of lung epithelial cells are regulated by androgen-signaling [62]. Additionally, NRP1 is a co-receptor of several tyrosine kinases responsible for controlling important cellular functions and pathways, including immune response, angiogenesis, cell survival, migration and invasion, and vascular biology [63]. NRP1 is highly expressed in the respiratory and olfactory epithelium [18], which can explain the SARS-CoV-2 infectivity and spreading through both the lungs and olfactory bulb. Eventually, it can spread into the central nervous system (CNS) [64], where NRP1 is involved in axonal guidance and pruning, mainly through its interaction with Semaphorin-3A (SEMA3A) [65,66]. Our data indicate that pseudoviral entry is aligned with the expression of NRP1 levels. This seems to substantiate the previous observations showing that NRP1 plays a major role for SARS-CoV-2 entry in certain cell types [16]. Therefore, it is crucial to characterize the infection susceptibility of specific neuronal cellular subsets that differ in molecular, morphological, connectional, and functional properties [67,68]. The regulation of NRP1 expression is unclear, but in certain cell types, it seems dependent on cellular activation through the IL-6/STAT3 and Wnt/β-catenin pathways [69,70,71].

Here, we demonstrated higher expression of both NRP1 and TMPRSS2, and a slight increase of ACE2 in RA-differentiated neuronal cells. This, in turn, allowed for the binding of the Spike protein, with subsequent increased fusion and entry into the differentiated neuronal cells as compared to the undifferentiated cells. Similar results were obtained by analyzing the expression of the surface molecules and the viral entry in HUVEC cells. Though our results need to be confirmed in vivo, based on our data, it is conceivable that other stimuli implicated in cellular differentiation [72] could contribute to the increased expression of molecules that facilitate SARS-CoV-2 entry into different subpopulations of neuronal cells, as well as could determine whether this could result in productive viral infection. The Spike protein binding to the several surface cellular proteins involved in fusion and entry, namely NRP1, ACE2, and TMPRSS2, as observed in both RA-differentiated neuronal cells and HUVEC, most likely would result in transducing receptor-mediated signal(s). How this signal may affect cell function needs to be elucidated. Finally, in light of our data, further studies should assess the possibility that recently emerged variants of interest and variants of concern (https://www.cdc.gov/coronavirus/2019-ncov/cases-updates/variant-surveillance/variant-info.html accessed on 31 August 2021), with different amino acid substitutions in the Spike protein, could result in different viral infection capabilities and functional dysregulation of particular subsets of neuronal cell populations. To better understand the clinical relevance and implications of our findings for diagnosis and treatment, samples collected from infected patients at different time points and with different Spike variants should be analyzed. Neuronal cells and endothelial cells are potential target for SARS-CoV-2 infection and the interaction of the Spike viral protein with these cells may cause their dysregulation. In addition, by interacting with all the different types of neuronal cells, endothelial cells participate in the regulation of molecular and cellular trafficking, including the circulation of viruses. Characterizing these stimuli and the cell subpopulations with variable inducible levels of viral co-receptor expression may help to advance our understanding of one of the many effects of SARS-CoV-2 in positive patients, namely the variety of neurological symptoms associated with the great majority of COVID-19 long-hauler patients [73,74].

## 4. Materials and Methods

### 4.1. Cell Culture

The SH-SY5Y cell line was obtained from ATCC (CRL-2266). Cells were cultured in a humidified incubator at 37 °C in 5% CO_2_ in F12 + EMEM (1:1) medium containing 10% fetal bovine serum (FBS), 100 U/mL of penicillin, and 100 U/mL of streptomycin. SH-SY5Y were treated with RA (10 µM) for 7 days [75]. RA was dissolved in ethanol, as indicated by the manufacturer (Sigma-Aldrich, St. Louis, MO, USA). Briefly, cells were seeded in T25 (or T75) flasks at a concentration of about 10^6^ cells/mL and RA was added at the beginning of the differentiation process. The medium was replaced every 2–3 days, with a concomitant addition of RA. Over time, the cells were observed under a direct light microscope to verify the progress towards differentiation into an elongated neuronal-like phenotype, as evidenced by a decreased amount of cell-body clumping and an extension of numerous thin, branched neuritic processes that often connect to neighboring cells. Cells treated with ethanol did not show any level of differentiation, particularly as they lacked neurite and neurofilaments, as well as synaptic vesicle recycling, and their proliferation rate was normal [60]. All these characteristics were similar to the ones observed in the SH-SY5Y cells not differentiated with RA, which was used as the control. Once the RA-treated SH-SY5Y cells reached their fully differentiation, all the procedures described later in this section (flow cytometry, RNA analysis, and confocal imaging) were conducted in absence of RA.

Human umbilical vein endothelial cells (HUVECs) from a single donor were obtained from Lonza (C2517A). Cells were cultured in a humidified incubator at 37 °C in 5% CO_2_ in EBM Basal Medium and with EGM Endothelial Cell Growth Medium Supplements, as required for the growth of endothelial cells (CC-3124).

### 4.2. Flow Cytometry

Cells were plated at a density of 3 × 10^5^ cells/well, in 6-well plates. After 4 days of culture, cells (including non-SH-SY5Y-differentiated and RA-differentiated SH-SY5Y cells, as well as HUVECs) were washed, detached, and resuspended in flow-staining buffer (PBS plus 2% FBS). Cells were stained using the following antibodies: anti-Neuropilin-1 Pe-conjugated, anti-ACE2 APC-conjugated (both from R&D Systems, Minneapolis, MN, USA), and anti-TMPRSS2 (H4) Pe-conjugated antibody (from Santa Cruz Biotechnology, Dallas, TX, USA). After 30 min of incubation at 4 °C, the cells were washed twice before the flow cytometry analysis with the Attune NxT Flow Cytometer (Thermo Fisher Scientific, Waltham, MA, USA). At least 30,000 cells were acquired for each experiment and the data were analyzed using FlowJo v10.8 (BD, Biosciences, San Jose, CA, USA). The positive populations were identified as cells that expressed specific levels of fluorescence activity above the non-specific auto fluorescence of the isotype control.

### 4.3. mRNA Isolation

Cells were washed with ice cold PBS and the pellets were resuspended in 100 µL of PBS. Total RNA was isolated using the miRVana microRNA isolation kit (Thermo Fisher Scientific, Waltham, MA, USA). RNA was eluted in 50 μL of RNase free-water and stored at −80 °C. The 260/280 and 260/230 ratios of the absorbance values were used to assess the purity of RNA using a Nanodrop ND-1000 spectrophotometer (Thermo Fisher Scientific, Waltham, MA). A 260/280 ratio of the ~2.0 and 260/230 ratio in the range of 2.0–2.2 was accepted as “pure” for RNA.

### 4.4. mRNA Quantification RT-qPCR

For the analysis of the target gene expression, 250 ng of mRNA was reverse-transcribed using the iScript Advanced cDNA Synthesis Kit for RT-qPCR (Bio-Rad Laboratories, Hercules, CA, USA) following the manufacturer’s instructions. Gene expression levels were detected with the following real-time PCR probe assays:

- NRP1 (qHsaCIP0039083) from Bio-Rad Laboratories, Hercules, CA, USA;
- ACE2 (qHsaCEP0051563) from Bio-Rad Laboratories, Hercules, CA, USA; and
- TMPRSS2 (Hs01122322_m1) from Thermo Fisher Scientific, Waltham, MA, USA.

Amplifications were performed in 12 μL of reaction mixture containing the 1X SsoAdvanced Universal Probes Supermix (Bio-Rad Laboratories, Hercules, CA, USA). The relative gene expression was calculated using reference standard curves for each target gene [76]. These were obtained by amplifying known positive gene DNA templates, each one carrying the sequence of NRP1, ACE2, or TMPRSS2. These positive controls were supplied by manufacturers and diluted by 10-folds from 20 million to 20 copies/µL following manufacturer’s instructions. The copy number of the mRNA of each gene per µg of the total RNA was calculated and indicated in the graphs. Aliquots of positive controls were prepared in distilled water and stored at −20 °C. Water was included for each of the amplifications as negative controls. qPCR amplification of both the samples and the respective positive control aliquots was carried out in parallels under the following conditions: 94 °C for 30 s of the initial denaturation step for 1 cycle; followed by 15 s of denaturation at 95 °C; and 30 s of annealing/extension at 60 °C for 40 cycles. All the reactions were carried out using the Bio-Rad CFX384 Real-Time PCR Detection System (Bio-Rad Laboratories, Hercules, CA, USA).

### 4.5. Infection, Immunofluorescence Staining, and Confocal Image Acquisition

For the infection experiments, we used the following pseudoviruses composed by the VSV backbone and expressing the Spike protein from SARS-CoV-carrying the D614G mutation (PV-SARS-CoV-2-S-(D614G)-VSV-△G-mCherry; BrainVTA, Wuhan, China). Cells, both non-differentiated and RA-differentiated, were plated in 8-well chambers (Nunc) with 1.0 borosillicate glass and at a concentration of 5 × 10^4^ cells/well; they were maintained in culture for 3 days, then infected for 24 h with about 2 × 10^5^ IFU/well, and finally washed in PBS 1X before fixation. The nuclei of the cells were stained with DAPI (20 µg/mL) immediately before imaging acquisition. Two laser lines of 405 nm (blue, for nuclei) and 561 nm (red, for pseudovirus particles) wavelengths were used (Carl Zeiss LSM 800 confocal system). Blue and red signals were separated by a dichroic beam splitter and were further acquired using a Gasp detector. A Plan-Apochromat (40×/1.2 water DIC objective) was used to visualize two-colored cell samples. All the parameters used in the confocal microscopy were consistent in each experiment, including the laser excitation power, detector, and offset gain. Software Zen Blue was used to generate original images and to collect z-stacks/the ORTHO mode (at a 0.5 micron size of sample slices). Negative control samples (non-infected cells) were stained/imaged with the same conditions as described above and used for the background calculation in the image analysis procedures. Optics instruments and software were obtained from Carl Zeiss, Germany. All the images were acquired under the same instrumental settings. The signal-to-noise ratio was assured by averaging data for every single image acquired. The saturated signal issue was avoided by using the software-controlled range. The total intensity of the sample was measured and averaged among all images per each set in order to assure the statistics.

### 4.6. Quantification of Cells Permissive to SARS-CoV-2 Spike-Mediated Viral Entry

The images acquired through the confocal system were analyzed to calculate the average intensity of the mCherry signal, the number of positive (infected) cells, and the intensity of the signal/positive cells. With the average intensity sum of the signal, we defined the sum of all positive intensities within the measured clusters; with the percentage of positive cells, we defined the number of cells associated with a red signal over the number of total cells; and with the average intensity sum of the signal/positive cells, we defined the intensity sum over the number of positive cells. During the acquisition process, we took 10 different fields for each well, and for each cluster of cells, we measured the intensity of the red positive signal (red pixels) and the number of infected cells.

### 4.7. Statistical Analysis

All statistical analyses were performed using the software GraphPad Prism v9 for Windows (GraphPad Software, San Diego, CA, www.graphpad.com). For single pairwise comparisons, statistical significance was determined by two-sided unpaired Student’s *t*-test with Welch’s correction for unequal variance. For multiple pairwise comparisons, a one-way analysis of variance (ANOVA) with Welch’s correction was conducted to identify significant differences within groups. A *p*-value of less than 0.05 was considered statistically significant.

## Supplementary Materials

The following are available online at https://www.mdpi.com/article/10.3390/v13112193/s1. Figure S1. (A–B) Undifferentiated SH-SY5Y cells cultured in complete growth medium. (C–D) RA-differentiated SH-SY5Y cells, cultured for 7 days with RA (10 µM). SH-SY5Y undergo profound morphological changes during the RA-treatment. Arrows indicate long axons-like structures developed during differentiation.
