# Supplementary material for: Comparison of SARS-CoV-2 Receptors Expression in Primary Endothelial Cells and Retinoic Acid-Differentiated Human Neuronal Cells
Source: Viruses. 2021 Oct 30;13(11):2193. doi: 10.3390/v13112193 (PMC8620655; doi:10.3390/v13112193)
Supplement: Supplementary file 1 [file viruses-13-02193-s001.zip › viruses-1383860 - legends supp.pdf]

viruses

MDPI

Supplemental material legends

Figure S1. (**A**-**B**) SH-SY5Y cells cultured in complete growth medium. (**C**-**D**) RA-differentiated SH-SY5Y cells, cultured for 7 days with RA (10 µM). SH-SY5Y undergo profound morphological changes during the RA-treatment. Arrows indicate long axons-like structures developed during differentiation.

Figure S2. Negative controls for SH-SY5Y cells background fluorescence: (**A**) SH-SY5Y cells and (**B**) RA-differentiated SH-SY5Y cells. SH-SY5Y cells were acquired by confocal microscopy. DAPI staining was used for nuclei detection. Scale bars: 10 µm.

Figure S3. Confocal images of SH-SY5Y cells infected with PV-SARS-CoV-2-S-(D614G)-VSV-ΔG-mCherry (D614G mutation) pseudovirus. (**A**) The collected Z-stacks of the corresponding gallery of images are shown, each presenting a 0.5-µm-thick slide. Scale bar: 10µm. (**B** and **C**) show a corresponding constructed 3D presentation and ortho mode of the protein expression.

Figure S4. Negative controls for HUVEC cells background fluorescence. HUVEC cells were acquired by confocal microscopy. DAPI staining was used for nuclei detection. Scale bars: 20 µm.

Figure S5. Confocal images of HUVEC cells infected with PV-SARS-CoV-2-S-(D614G)-VSV-ΔG-mCherry (D614G mutation) pseudovirus**.** **(A)** The collected Z-stacks of the corresponding gallery of images are shown, each presenting a 0.5-µm-thick slide. Scale bar: 10µm. **(B and C)** show a corresponding constructed 3D presentation and ortho mode of the protein expression.
