# Supplementary figures and images for: Comparison of SARS-CoV-2 Receptors Expression in Primary Endothelial Cells and Retinoic Acid-Differentiated Human Neuronal Cells
Source: Viruses. 2021 Oct 30;13(11):2193. doi: 10.3390/v13112193 (PMC8620655; doi:10.3390/v13112193)

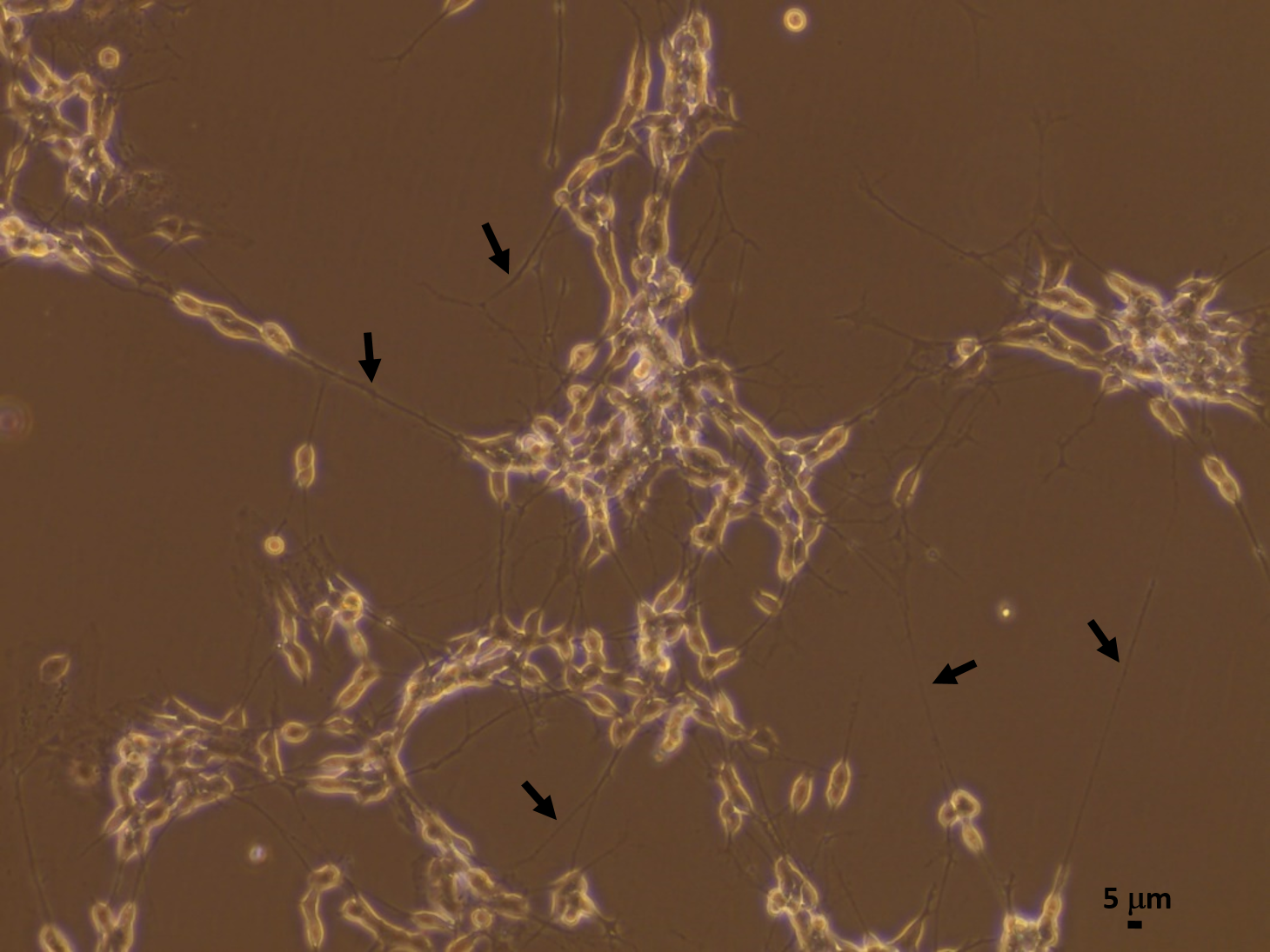

Supplement: Supplementary file 1 [file viruses-13-02193-s001.zip › Fig.S1C.png]

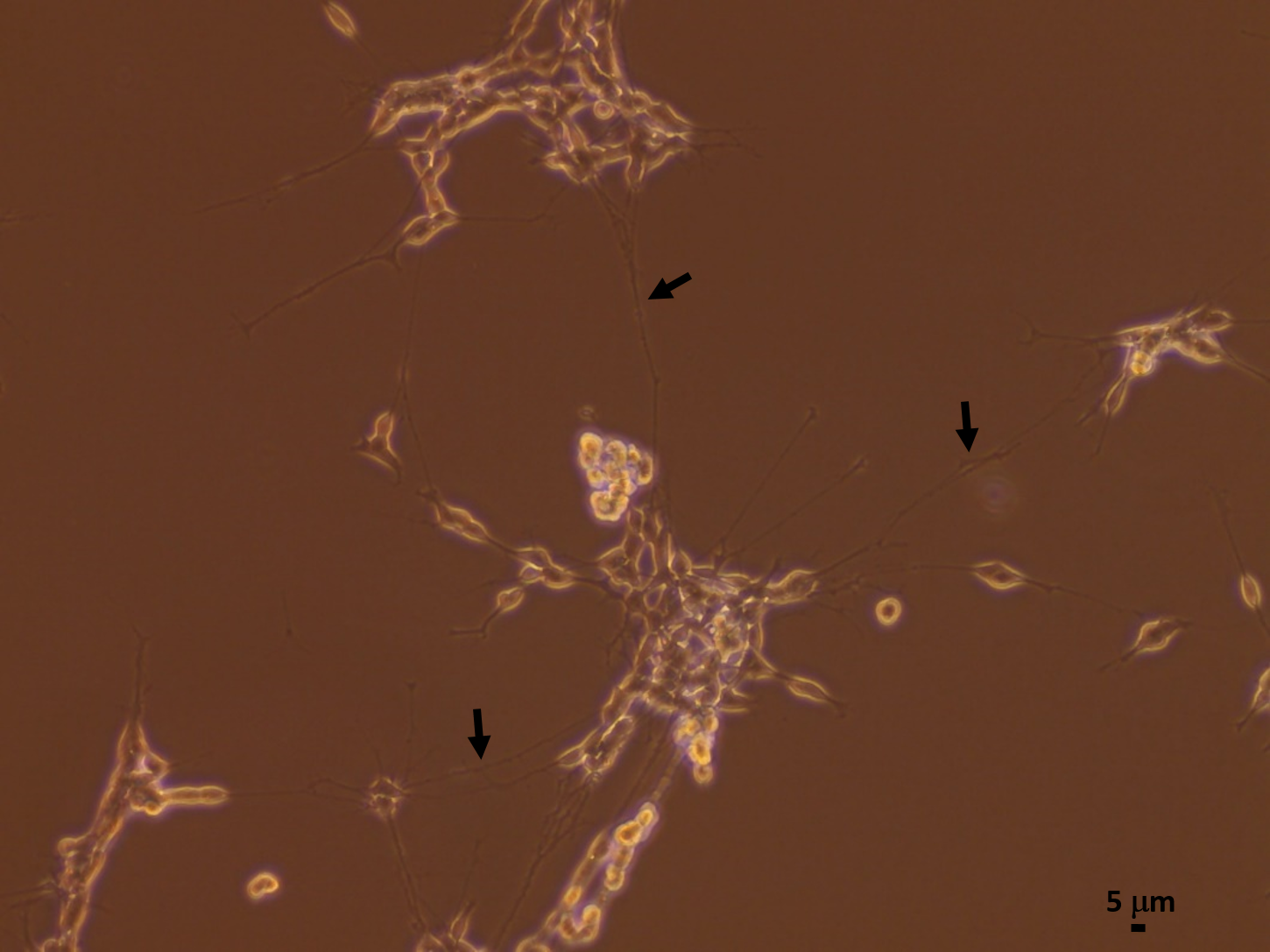

Supplement: Supplementary file 1 [file viruses-13-02193-s001.zip › Fig.S1D.png]

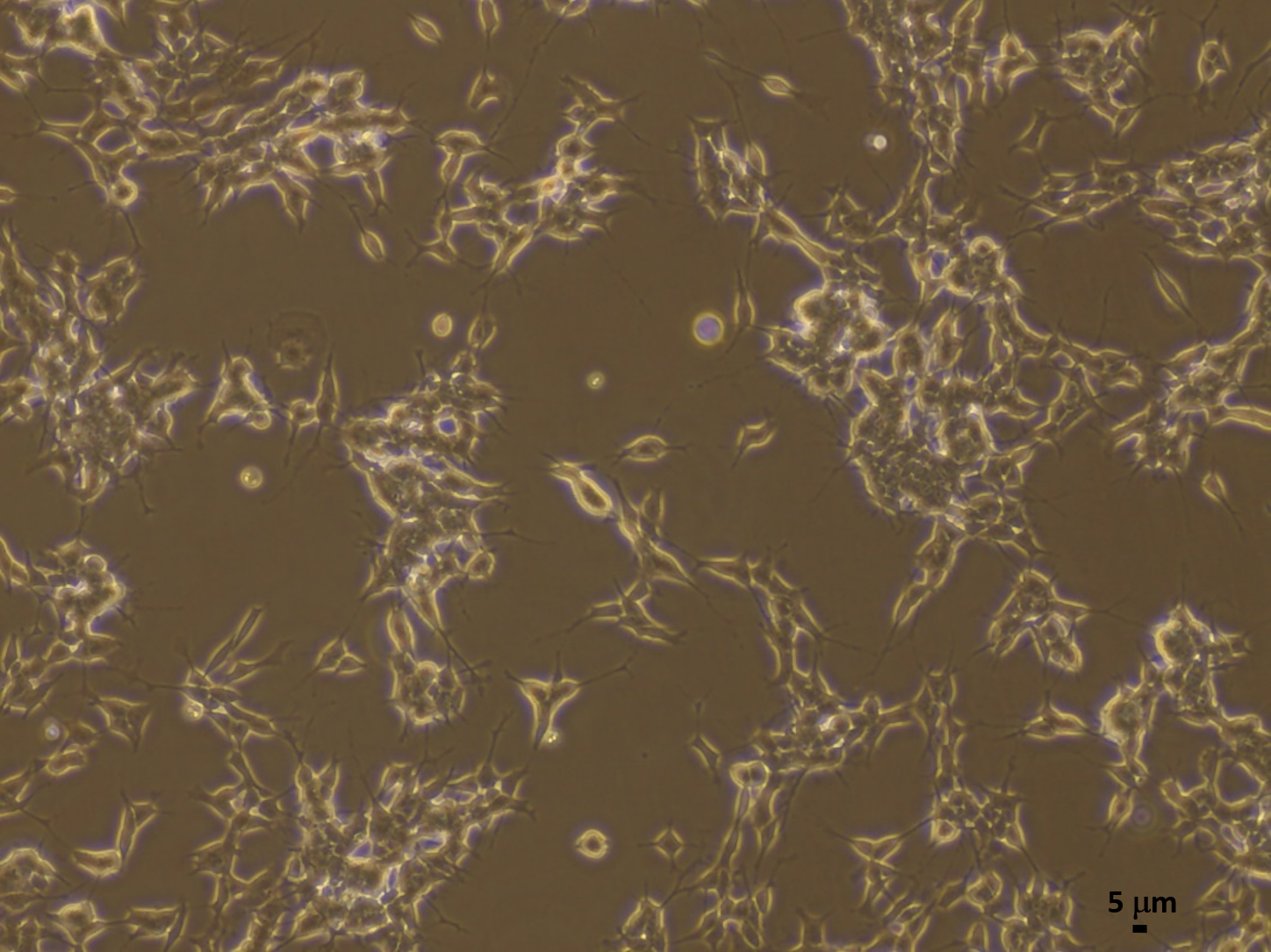

Supplement: Supplementary file 1 [file viruses-13-02193-s001.zip › FigS1A.png]

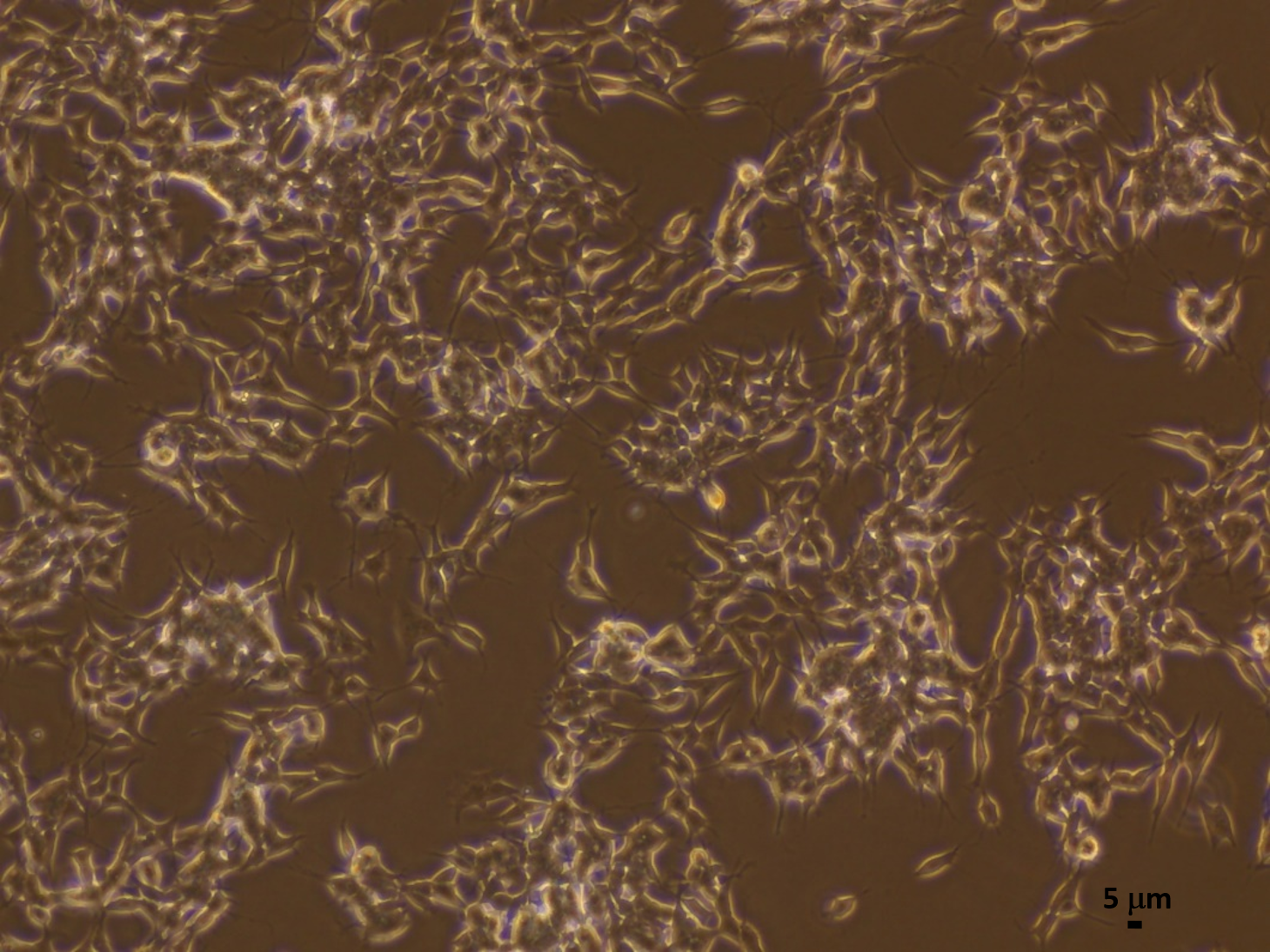

Supplement: Supplementary file 1 [file viruses-13-02193-s001.zip › FigS1B.png]

**A**

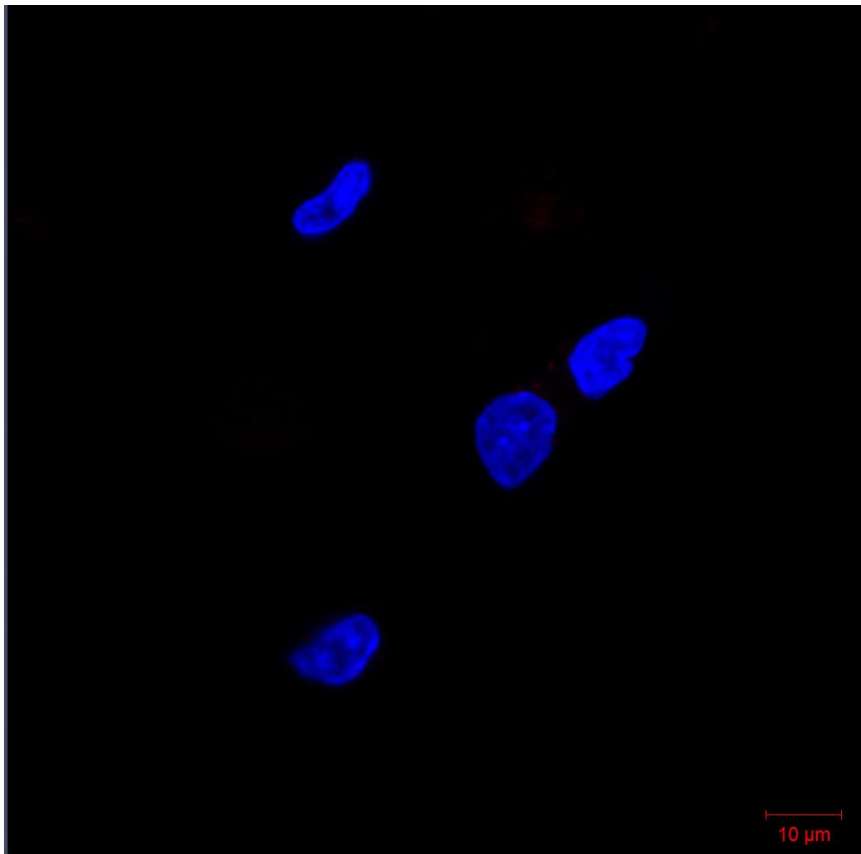

**Not treated**

**B**

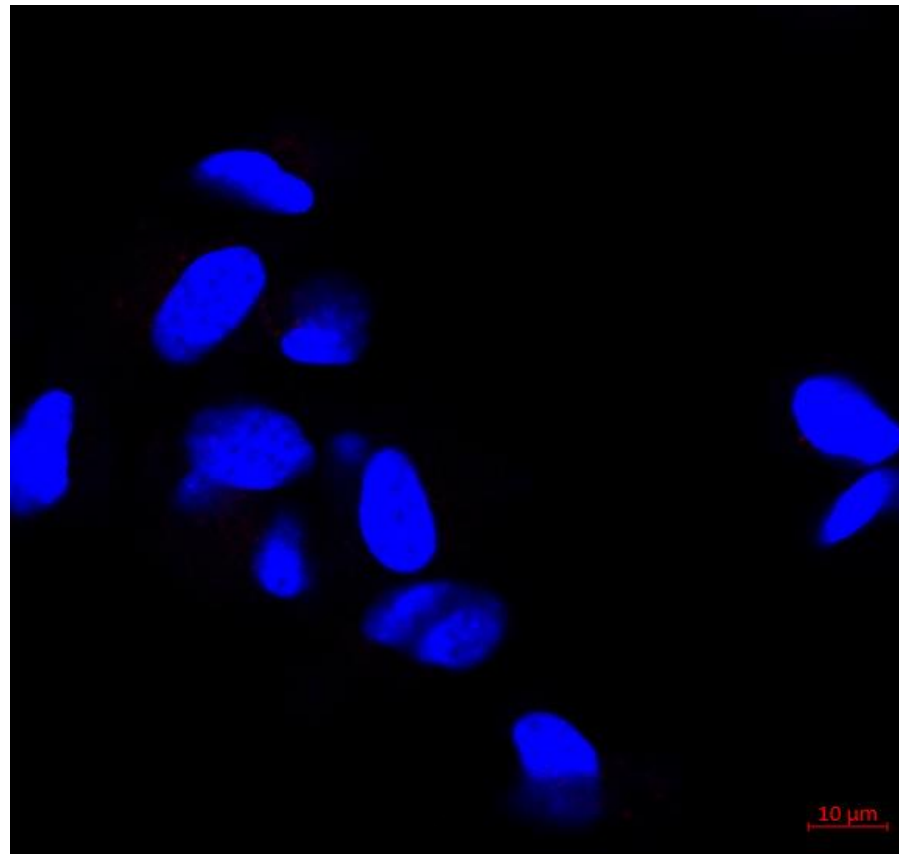

**RA-treated**

Supplement: Supplementary file 1 [file viruses-13-02193-s001.zip › FigS2.pdf]

**A**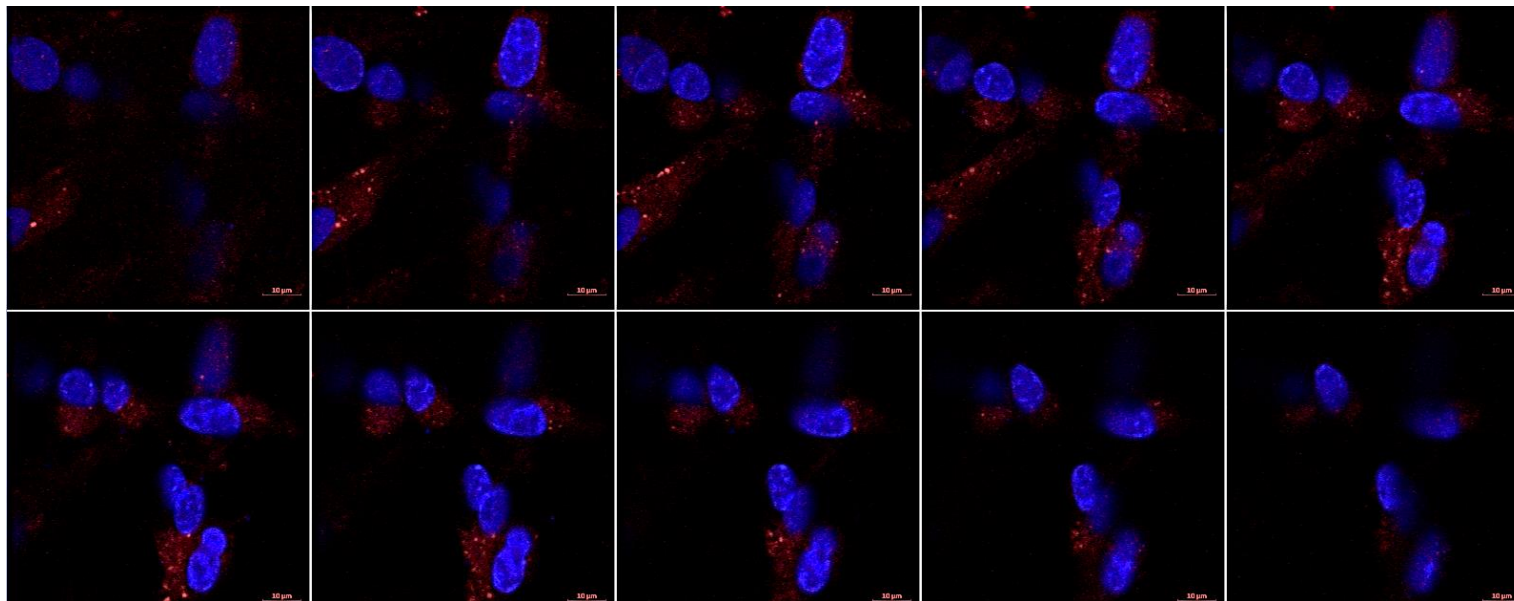**B**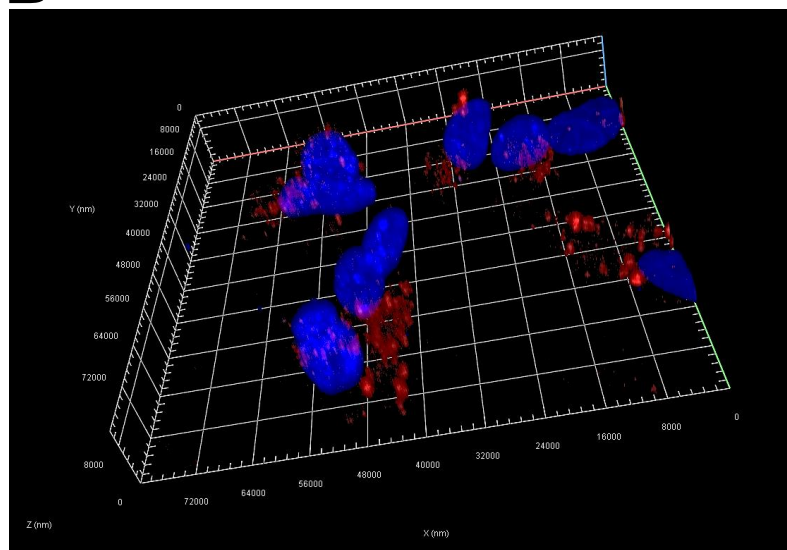**C**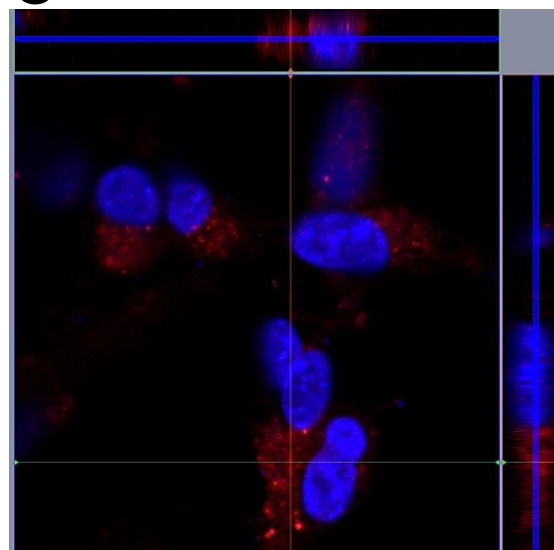

Supplement: Supplementary file 1 [file viruses-13-02193-s001.zip › FigS3.pdf]

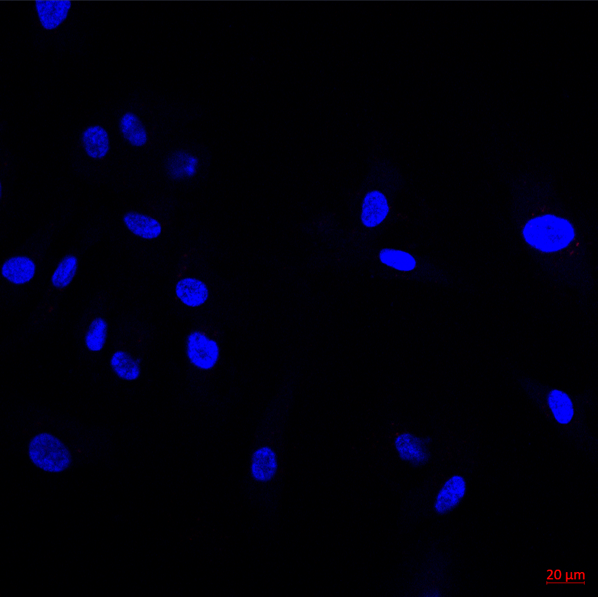

Supplement: Supplementary file 1 [file viruses-13-02193-s001.zip › FigS4.png]

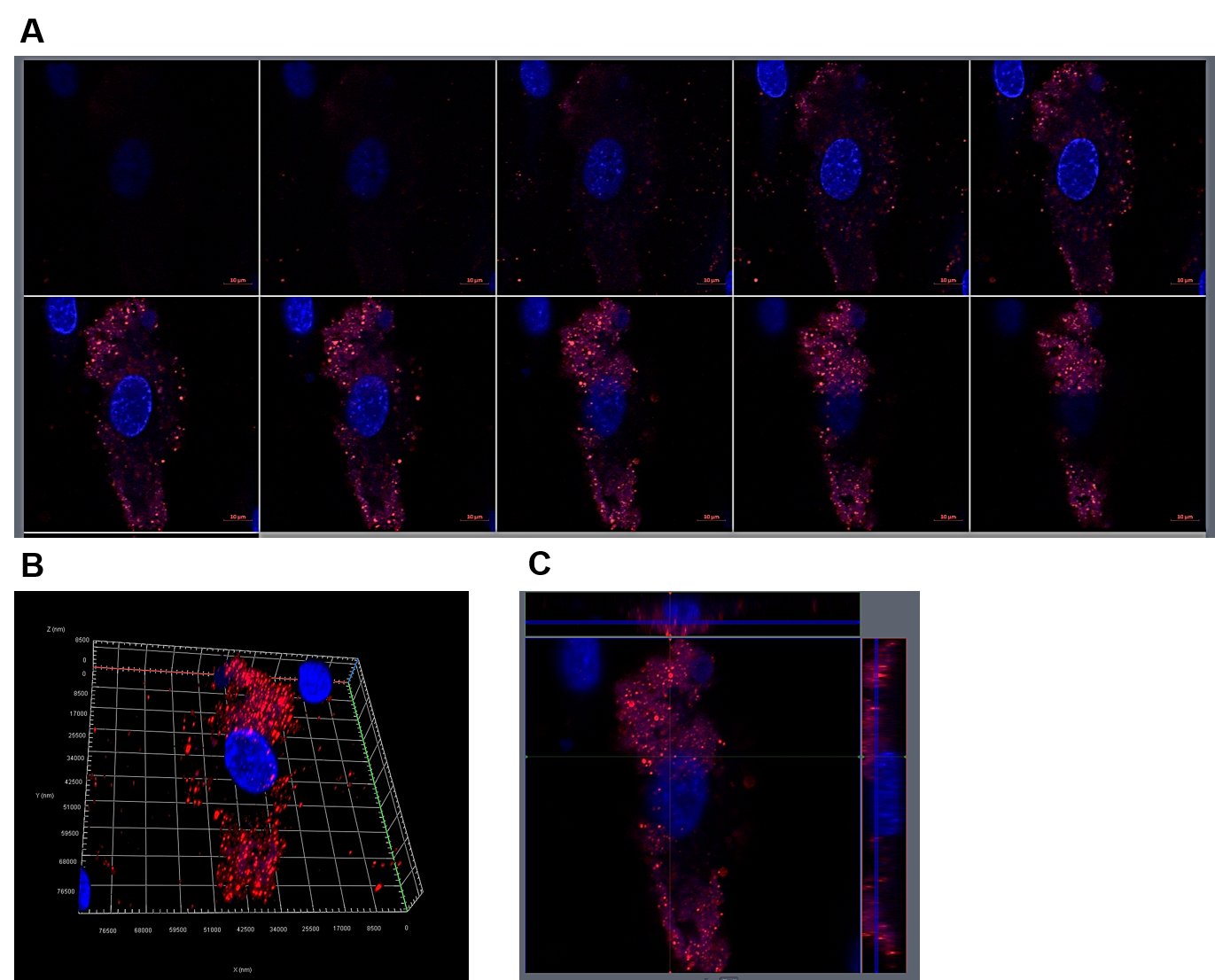

Supplement: Supplementary file 1 [file viruses-13-02193-s001.zip › FigS5.png]
